# Primary Cilia and Calcium Signaling Interactions

**DOI:** 10.3390/ijms21197109

**Published:** 2020-09-26

**Authors:** Hannah Saternos, Sidney Ley, Wissam AbouAlaiwi

**Affiliations:** Department of Pharmacology and Experimental Therapeutics, University of Toledo Health Science Campus, Toledo, OH 43614, USA; hannah.saternos@rockets.utoledo.edu (H.S.); sidney.ley@rockets.utoledo.edu (S.L.)

**Keywords:** primary cilia, calcium signaling, fluid shear, mechanosensation, chemosensation

## Abstract

The calcium ion (Ca^2+^) is a diverse secondary messenger with a near-ubiquitous role in a vast array of cellular processes. Cilia are present on nearly every cell type in either a motile or non-motile form; motile cilia generate fluid flow needed for a variety of biological processes, such as left–right body patterning during development, while non-motile cilia serve as the signaling powerhouses of the cell, with vital singling receptors localized to their ciliary membranes. Much of the research currently available on Ca^2+^-dependent cellular actions and primary cilia are tissue-specific processes. However, basic stimuli-sensing pathways, such as mechanosensation, chemosensation, and electrical sensation (electrosensation), are complex processes entangled in many intersecting pathways; an overview of proposed functions involving cilia and Ca^2+^ interplay will be briefly summarized here. Next, we will focus on summarizing the evidence for their interactions in basic cellular activities, including the cell cycle, cell polarity and migration, neuronal pattering, glucose-mediated insulin secretion, biliary regulation, and bone formation. Literature investigating the role of cilia and Ca^2+^-dependent processes at a single-cellular level appears to be scarce, though overlapping signaling pathways imply that cilia and Ca^2+^ interact with each other on this level in widespread and varied ways on a perpetual basis. Vastly different cellular functions across many different cell types depend on context-specific Ca^2+^ and cilia interactions to trigger the correct physiological responses, and abnormalities in these interactions, whether at the tissue or the single-cell level, can result in diseases known as ciliopathies; due to their clinical relevance, pathological alterations of cilia function and Ca^2+^ signaling will also be briefly touched upon throughout this review.

## 1. Introduction

Adaptation has long been thought to be the key evolutionary development required for life to exist. We owe our longevity as a species to our ability to adapt to changes in our external environment. Before large-scale adaptations can occur, cells must first possess the ability to adapt and respond to external stimuli in the environment. Because cells face a barrage of minute chemical and physical stimuli on a perpetual basis, cells must precisely signal in order to correctly respond to stress. These signals require messengers whose concentrations can change quickly depending on time and location. Calcium cations (Ca^2+^) are one such messenger and are involved in many different cellular processes and translating external stimuli into intracellular signaling cascades, as well as having a near-ubiquitous role in the actions of the diverse cellular processes initiated. The success of this complex role may, in part, be due to the heterogenic distribution of Ca^2+^ in areas called “Ca^2+^ microdomains” found in resting and stimulated cells [1,2].

Cilia are hair-like organelles that protrude from the apical surface of mammalian cells and fall into two broad categories: motile and non-motile. Motile cilia possess the dynein motor complexes needed to move, while non-motile ones do not; however, both house a 25 μm diameter cytoskeletal scaffold known as the axoneme [3], which comprises hundreds of proteins and houses nine peripheral microtubule doublets made up of A and B tubules. These tubules surround a varying amount of microtubules which determines a cilium’s structure classification; the presence of two microtubules results in a 9 + 2 pattern classification, while a lack of microtubules results in a 9 + 0 pattern [3]. Non-motile cilia, known as primary cilia, have a 9 + 0 structure and exist as monocilia on the surface of cells. Some motile cilia contain a 9 + 2 pattern and exist in clusters on cells known as multiciliated cells, while other motile cilia, known as nodal cilia, have a 9 + 0 structure and exist as solitary monocilia on cell surfaces [4]. For motile cilia, the presence or absence of the central pair leads to significant movement pattern differences. The 9 + 2 structure commonly moves in a wave-like motion to generate fluid flow; an example of which is ependymal cilia. The 9 + 0 structured nodal cilia commonly move in a rotary or corkscrew motion, as seen in the nodal cilia present in the nodes responsible for organ patterning in embryonic development [5], and also as seen in flagella, where this motion is used for propulsion [6,7]. The primary cilium is quickly gaining fame as a signaling powerhouse of the cell, and as such shall be the main cilium of focus for this review.

Although primary cilia appear to be a continuous extension of the cellular membrane, they have a distinctly unique membrane composition compared to the rest of the cell. Ciliary membranes have a vast array of specifically localized receptors and channels making the detection, transmission, and translation of external mechanical or chemical stimuli their principal function within the cell [8,9]. High levels of Ca^2+^-permeable channels, such as polycystin 2 (PC-2) and transient receptor potential cation channel subfamily V member 4 (TRPV4), are present on both the ciliary membrane and basal body; the intracellular anchor point the cilium originates from [10]. It is generally believed that an influx of extracellular Ca^2+^ into the primary cilia precedes a rise in cytosolic Ca^2+^; however, the exact mechanism by which this happens remains unknown [11,12,13,14,15,16]. To further complicate things, there may not be a uniform mechanism explaining ciliary Ca^2+^ signaling but one that is instead cell-type specific, as the type of Ca^2+^ channels and ciliary activation pathways vary by cell type. For example, PC-2 and TRPV4 have been localized to the primary cilia in both the kidney and bone cells, but research suggests a polycystin-mediated Ca^2+^ influx in the kidney and a TRPV4-mediated influx in bone [17,18,19,20]. In this review, we aim to touch on findings related to the signal transduction mechanisms in the cilia and Ca^2+^-dependent biologic processes that may be mediated through ciliary signaling.

## 2. Calcium and Ciliary Signal Transduction: Sensory Function and Cilia Structure

The sensory role of the primary cilium is highly specialized depending upon the tissue or organ system it is localized in. Well-known examples of their specialized roles include mechanosensitive cilia found in blood vessels, kidney, and bone; chemosensitive cilia in the nose; and recently, new evidence has suggested an electrosensitive role of primary cilia in the nervous system [21,22,23]. To effectively translate such a diverse range of extracellular signals, ciliary protein and receptor composition, as well as cilia structure, all come together to contribute to context-appropriate signaling [24].

### 2.1. Mechanosensation

**Figure 1:**
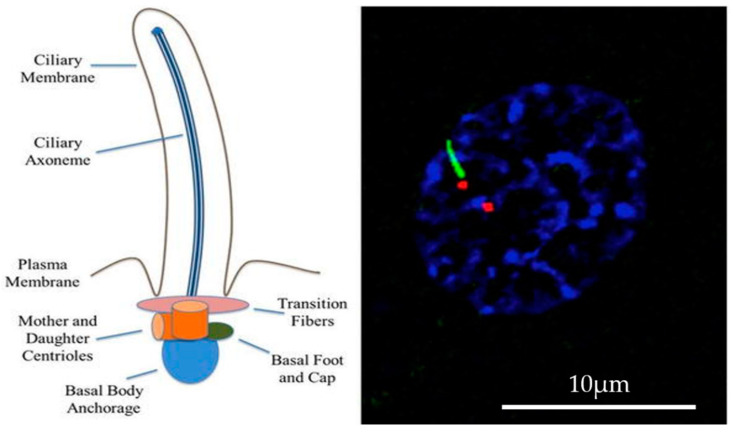
Primary cilia structure. The axonemes of primary cilia are anchored on the basal body and encapsulated within the ciliary membrane. The ciliary membrane is one continuous extension of the plasma membrane. The basal body is composed of the mother and the daughter centrioles, as well as some transition fibers that anchor the basal body to the cell membrane. The ciliary membrane houses specific membrane and protein receptors, all of which facilitate proper cilia signaling (left panel). Primary cilia that are found on vascular endothelial cells are identifiable by an immunofluorescence technique with antibody against acetylated α-tubulin (green) labeling primary cilia, and pericentrin (red) labeling the centriole or basal body. The nucleus is counterstained with DAPI (blue) to label DNA (right panel). Left panel is adopted with permission from ref. [27].

The primary cilia structure is highly conserved, serving as an antenna for extracellular information and converting mechanical or chemical stimuli into electrical signals the cells can interpret [24]. These electrical signals are often driven by changes in Ca^2+^ levels that initiate signaling pathways within the cell. Generally, non-motile primary cilia are said to have a 9 + 0 structure, or 9 doublet microtubules arranged in a circle and emanating from an intracellular anchor point called the basal body (Figure 1). However, a study by Sun et al. showed evidence of a structural deviation in mechanosensitive cilia, specifically those involved in fluid flow sensation [25]. Kidney epithelial and vascular endothelial cell cilia have very similar roles; both detect fluid motion through either kidney tubules or blood vessels to maintain tissue homeostasis and proper function in their respective organ system. The mechanosensation abilities of these cilia are passively mediated by fluid-induced deflection which initiates specific downstream signaling cascades [26]. Three-dimensional models and advanced imaging techniques show that while the concentric structure of the axoneme is maintained, the diameter of the cilium tapers towards the distal end of the cilia due to a reducing number of microtubules; from a 9 + 0 structure to 7 + 2, 5 + 2, and then finally 3 + 0. This significant change in structural configuration contributes to the elasticity of the cilium and its bending properties [25,26].

**Figure 2:**
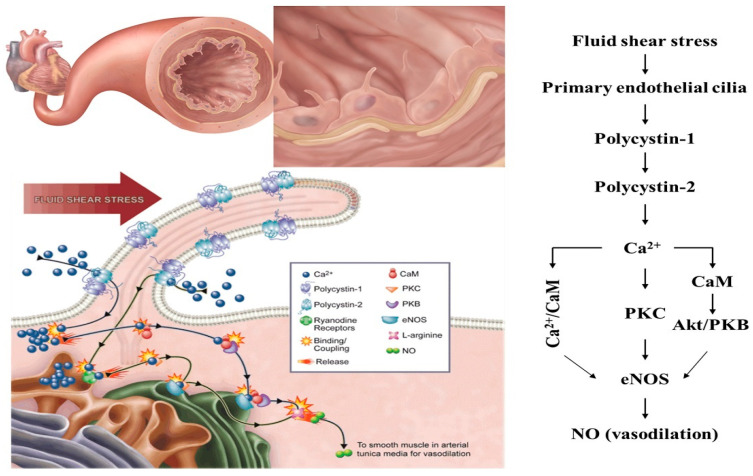
Primary cilia activation by fluid shear stress and nitric oxide (NO) signaling in the vascular endothelia. Left panel: Primary cilia bending in response to fluid flow-generated shear stress. Subsequent biosynthesis of and release of nitric oxide (NO) is also shown. Right Panel: The production and release of NO occurs due to activation of primary cilia within endothelial vasculature. When cilia experience shear stress, the mechanosensory polycystin complex activates, which initiates the synthesis and release of NO. The resultant biochemical cascade involves an extracellular calcium (Ca^2+^) influx, followed by the activation of multiple Ca^2+^-dependent proteins, including calmodulin (CaM), protein kinase C (PKC), and AKT/PKB, which in turn trigger endothelial nitric oxide synthase (eNOS), and NO is produced and released. Figure is adopted with permission from ref. [30].

While it is unclear how the mechanics of primary cilia contribute to the mechanosensitive properties of cilia, there is mounting evidence suggesting that the passive bending of the cilia in response to fluid shear stress activates Ca^2+^ channels and mechanosensitive receptors on the ciliary body and causes strains on the internal structures in the cell, thereby collectively regulating the mechanosensitive response (Figure 2) [12,19,24]. Ciliary bending in response to shear stress appears to be influenced by both length and flexural rigidity or stiffness of the axoneme; the degree of ciliary bending has also been shown to regulate the strength of downstream signaling cascades such as Wnt, platelet-derived growth factor (PDGF), and hedgehog (Hh) [12]. Studies using renal cell lines and rat tail tendon cells show that as intracellular Ca^2+^ levels are reduced, the cilia length increases, suggesting an inverse relationship between cilia length and mechanosensory function [24,28,29]. More recent computational models of cilia report that longer, more flexible cilia equated to an increase in strain on the cilium structure and internal cellular components, and that stiffer cilia and/or shorter cilia had smaller deflections, leading to less strain. This suggests a direct relationship between cilia length and flexibility, and mechanosensation [24]. Besschetnova et al. proposes a Ca^2+^-dependent mechanism for the regulation of cilia length, suggesting that reduced intracellular Ca^2+^ levels and elevated cAMP levels cause an increase in cilia length. The elongated cilia then create a negative feedback loop whereby the cilia bending decreases cAMP, increases intracellular Ca^2+^, and shortens the cilia, thereby decreasing mechanotransduction [28].

Ciliary mechanosensation is also regulated by mechanosensitive proteins that localize to the primary cilia, the polycystin complex being one of the more well-researched proteins. Arguably, the shift in viewpoint from cilia being vestigial to having an important function in the body began with the discovery of polycystin 1 and 2 and their connection to polycystic kidney disease (PKD). Polycystin 1 (PC-1), a mechanosensitive transmembrane protein, and PC-2, a Ca^2+^-permeable cation channel, form a complex that localizes to the axoneme of primary cilia and has been shown to be important for mechanosensing and interpreting fluid shear stress in both renal tubules and blood vessels. Proper kidney function depends on regulated fluid flow through the nephrons and collecting ducts; this controls the glomerular filtration rate [31,32]. In renal cells, defects in the PC-1/2 complex, the primary cilia structure, or ciliary protein composition can lead to various kidney problems, including PKD [33,34,35,36,37]. Research on abnormal cilia mechano-function in the kidneys has centered around flow-induced changes in intracellular Ca^2+^; when either PC-1 or PC-2 is altered, the expected elevation in intracellular Ca^2+^ is abolished, thereby altering downstream signaling cascades and activating pro-cystogenic pathways. Interestingly, this has been observed in a cilia-less mutant renal cell line, orpk, suggesting that a loss of protein function or a loss of the cilia structure dysregulates Ca^2+^ entry into the cell and promotes aberrant tissue maintenance [38]. Similarly, cilia on vascular endothelial cells are also responsible for detecting fluid shear stress in order to maintain blood pressure. An increase in blood flow or volume increases the shear stress acting upon endothelial primary cilia, resulting in the production of nitric oxide (NO) which diffuses into the surrounding smooth muscle cells, resulting in vasorelaxation [39]. This response is, in part, due to the PC-1/2 complex which produces an increase in intracellular Ca^2+^, triggering the calcium/calmodulin complex (Ca^2+^/CaM) to activate endothelial nitric oxide synthase (eNOS) (Figure 2) [19,40,41]. Studies investigating PC-1’s exact mechanosensory abilities found that PC-1 knockout vascular endothelial cells failed to produce an increase in cytosolic Ca^2+^ and the accompanying NO biosynthesis and release. In an effort to confirm this as a role for ciliary polycystins specifically, cells lacking cilia but maintaining polycystin expression were also tested; the results showed that neither Ca^2+^ nor NO signals were produced at normal and high flow rates [41]. When tested, PC-2 knockout vascular endothelial cells also showed a reduction in both Ca^2+^ signaling and the resultant NO flux under shear stress conditions. When tested further in ex vivo studies using endothelial cells isolated from pkd2^−/−^ mouse arteries, the results were the same; a lack of response to fluid shear stress [19]. This revealed that not only are both ciliary PC-1 and PC-2 needed for cilia mechanosensation and mechanotransduction, but also that the PC-1/2 complex initiates the signaling cascade needed for Ca^2+-^dependent NO biosynthesis.

However, despite many studies supporting the model in Figure 2, the origin of the Ca^2+^ involved in secondary messaging within the cilium is still under debate. A study by Delling et al. showed that in cells bearing primary cilia, the Ca^2+^ wave originated out in the cytoplasm before propagating in the primary cilium [42,43], suggesting a lack of temporal resolution, leading to digital artifacts, which caused other researchers to misinterpret the Ca^2+^ mobilization [12]. While possible, Delling et al. did not subject cells to flow for the longer durations that previous groups required to examine peak Ca^2+^ flux, and therefore more studies are needed to follow up on this hypothesis [12]. If primary cilia are indeed not Ca^2+^-responsive mechanosensors, the origin of the mechanically induced Ca^2+^ waves is currently a mystery [43]. When cytoplasmic free Ca^2+^ increases, an increase in ciliary Ca^2+^ follows, which indirectly potentiates the activity of the ciliary PC-2 channel; if TRP channels are not involved in this process, other possible mechanosensitive proteins that do not elicit changes in ciliary Ca^2+^, such as G-protein-coupled receptor (GPCR) proteins, may be responsible [44,45].

### 2.2. Chemosensation

All primary cilia have a very diverse chemosensory role, housing a wide array of receptors and proteins for ligands, ranging from hormones to neurotransmitters, endogenous molecules, and even exogenous molecules [30,46,47,48]. Because the sheer number of known chemosensitive pathways would be impossible to cover, and downstream signaling would be unique to receptor activation, we will briefly touch on olfaction to highlight the receptor elements of primary cilia. Structurally, olfactory cilia localize to the olfactory knob of olfactory sensory neurons (OSN) [49]. Olfactory sensory cilia are responsible for the perception of smell; odors from our environment bind to GPCRs found on the cilia of OSN. Genetic analysis has shown that humans have about 400 GPCR-coding genes expressed in a specific pattern on olfactory cilia. Binding to any one of the GPCRs results in activation of ciliary cyclic nucleotide-gated channels (CNGs) that directly mediate the influx of Ca^2+^, depolarizing the neuron and transmitting the signal to the brain where it is interpreted accordingly [50,51]. Perturbations in the localization of adenylate cyclase III, CNGs, and Ca^2+^-activated chloride channels, or in the cilia structure itself can lead to an impaired sense of smell (anosmia) [49,50,51]. While anosmia was originally associated with Bardet-Biedl Syndrome and Leber congenital amaurosis, two known ciliopathies, it is now known to be associated with a subset of ciliopathies that have mutations in Cep290 or KIF17 [52].

### 2.3. Electrosensation

Whether or not primary cilia detect and transduce electrical stimuli is difficult to identify, as studies show results that are highly variable and dependent on the frequency of stimuli, the strength of the electrical field, and cell type [53,54]. To make matters more complicated, there are several mechanisms for electrical transduction within the cell. Of those transduction pathways, the opening of ion channels, including Ca^2+^ channels [54,55,56], and transduction through electromechanical forces [57,58] may involve primary cilia. However, current methods to investigate a ciliary role in electrosensation are limited to indirect measurements and mechanistic inferences, citing proteins typically associated with electrical signaling being localized to primary cilium or the removal of the cilia structure abolishing a normal response to electrical stimuli [59]. More direct measurements have been attempted using fluorescent Ca^2+^ indicators which report extracellular-dependent changes in intracellular Ca^2+^ levels suggestive of an electrical current being carried across the ciliary membrane [59]. While incredibly useful, this approach has difficulties surrounding the primary cilium’s diameter and length, which creates unique challenges in recording this type of transduction [59]. This is limitation is evident in a study that used electrical stimulation of human adipose stem cells to induce osteogenic differentiation; when exposed to an electrical field of 10mV, an upregulation in ciliary structural proteins was observed as well as intracellular Ca^2+^ oscillations. More interestingly, this study reported that knocking out *IFT88* (encoding for ciliary structural protein), or *PKD1* (encoding for PC-1), the stem cells lost their ability to respond to electrical field stimulation, as in differentiation and osteogenic markers, but an increase in cytoplasmic Ca^2+^ was still observed. This could be explained by either cytoplasmic Ca^2+^ being needed to precede cilia sensory function, enabling the cilia to sense the electrical stimuli, or a separate Ca^2+^ microdomain forming in the cilia that is distinct from cytosolic Ca^2+^. Together, this suggests that primary cilia have a critical role in translating Ca^2+^-induced cellular responses into electrical stimulation [21].

## 3. Cilia-Mediated and Calcium-Dependent Biological Processes

As stated previously, cells have evolved to invest much time and energy into controlling Ca^2+^ concentrations. The ability of Ca^2+^ to bind and initiate changes in protein shape and charge, which are important parameters in protein function, make it an ideal secondary messenger. Ca^2+^ carries out a vast array of complex cellular processes, and many different factors cause fluxes in intracellular Ca^2+^; hormones, growth factors, cytokines, and neurotransmitters all increase intracellular Ca^2+^, but due to the temporal and spatial nature of Ca^2+^, a cell is able to tailor its response to any specific hormone or stimuli [1,60]. Unlike more complex molecules, Ca^2+^ cannot be chemically altered. Thus, to exert control over Ca^2+^, cells must chelate, compartmentalize, or extrude it [1,2,60]. Much of the research currently available on Ca^2+^-dependent cellular actions and cilia are tissue-specific processes (Table 1); vasodilatation [10,19,41], osteogenesis [61,62,63,64], olfaction [63,64], and left-right asymmetry [62,64] all involve primary cilia and Ca^2+^ interplay. However, literature investigating the direct connection between primary cilia and Ca^2+^-dependent processes at the cellular level appears to be scarce. Thus, this section will focus on summarizing the available evidence for the parts they play together in some basic cellular activities and pathways (Table 1).

### 3.1. Cell Cycle

Although several connections to the cell cycle can be made through ciliary-localized proliferative pathways, the connection between primary cilia and the cell cycle lies in the dependence on the centrosome [76,77]. However, as both rely on the same organelle, they also are mutually exclusive processes that do not co-exist, and thus the cell is constantly teetering on which process to favor [76]. As the cell progresses through the cell cycle, Ca^2+^ levels fluctuate in a specific manner, modulating the cell’s proliferative responses [78]. In mammalian cells, both extracellular and intracellular Ca^2+^ is important for cell cycle progression; a depletion in either results in the cessation of division. Specifically, extracellular Ca^2+^ influxes are important during the G_0_/G_1_ phase and G_1_/S transition as well as during cytokinesis, suggesting that the highest levels of Ca^2+^ transients are important in cell cycle entry and exit. When Ca^2+^ enters the cell, its main intracellular receptor is calmodulin (CaM), the concentrations of which are also tightly regulated during cell division. Several studies have reported that overexpression of CaM shortens the G_1_ phase and accelerates proliferation; interestingly, cilia length has been shown to directly impact the time spent in this phase, with more evidence suggesting cells with shortened cilia enter S-phase more rapidly [76,79,80]. As a complex, Ca^2+^/CaM exerts its proliferative effect through the use of Ca^2+^/CaM-dependent kinases I, II, and IV. One way Ca^2+^/CaM helps coordinate entry into the cell cycle is through the centrosome cycle. For division to occur, the centrioles disengage and migrate to opposite poles in the cell, where they then duplicate, creating the two mitotic spindle poles. Studies show Ca^2+^/CaM and CaM-dependent protein kinase II (CaMKII) regulate the expression of centriolar coiled-coil protein of 110 kDa (CP110), in addition to several other transcription factors that are necessary for centrosomal duplication, correct spindle formation, and cytokinesis regulation [60,81]. Studies show the same growth factors initiating both ciliary reabsorption and cell cycle entry have also been shown to activate a series of ciliary-localized Ca^2+^ channels: TRPV2 (Transient Receptor Potential Cation Channel Subfamily V Member 2), PC-2, and TRPC1 (Transient Receptor Potential Cation Channel Subfamily C Member 1) [72]. The high density of Ca^2+^ permeable channels allows for an isolated rise in intraciliary Ca^2+^, creating a localized signaling pathway directly from the cilia, to the basal body, and then to the nucleus. Further evidence shows CaM and Ca^2+^/CaMKII localize to cilia and are downstream of the ciliary mechanosensory PC-1/PC-2 complex. As cilia bend, the activation of PC-1 triggers two signaling cascades. The first is through the activation of PC-2, enabling an extracellular Ca^2+^ influx and leading to the activation of Ca^2+^/CaM-dependent pathways. Secondly, the intracellular tail of PC-1 gets cleaved and translocated to the nucleus, where it regulates DNA transcription via genes related to proliferation [65,66,82,83].

### 3.2. Cell Polarity and Migration

The directional movement of cells to specific locations is fundamental for immune responses, tissue homeostasis, and many developmental processes [84,85]. The asymmetric distribution of organelles and molecules in the cell is generally what defines cell polarity and provides direction and orientation in the cell. Almost all cells have slight polarities; however, there are specific cell types where polarization is important for their function. For example, endothelial and epithelial cells typically have apical-basal polarity where the apical membrane faces towards a lumen and establishes their barrier function. Migratory cells such as leukocytes and fibroblasts have a defined front and rear, termed the leading and trailing edge respectively. In some mammalian cells, one of the early signs of planar cell polarity is the establishment of the cilia on the apical side of the cell [86]. Many pathways involved in cell migration have been associated with primary cilia such as Hh, Wnt, and transforming growth factor beta (TGFβ) [87,88,89,90]. Cellular polarization is necessary to coordinate directional migration and can also do so through the multifaceted role of Ca^2+^. In cell migration, Ca^2+^ is a player in sensing direction, cytoskeletal rearrangements, traction force, and the localization of focal adhesion molecules. Doyle et al. reported, in polarized cells, Ca^2+^ transients increased starting at the “front” of the cell and gradually moved towards the “rear.” In contrast, the observations in non-polarized cells showed an even distribution of Ca^2+^ throughout the cell [90]. These results parallel scratch-test studies done in fibroblasts where 30-60 minutes after a wound was created, primary cilia would reorient themselves around the wound edge, parallel to the direction of movement. This was not observed in non-migrating cells where the directional orientation of cilia was more random [91]. While the direct connection between cilia and Ca^2+^ in not yet fully understood, there is evidence to suggest that cilia, potentially through ciliary-dependent Ca^2+^ cascades, may stand as a point of reference for the coordinated movement of cells, interpreting and aiding in creating an environment with opposing and synergistic signals [92].

### 3.3. Neuronal Patterning

Neurons originate from proliferating cell progenitors in the neuroepithelium. The neuroepithelium forms the wall of the neural tube, the embryonic precursor to the central nervous system. Neuronal patterning is the process by which cells in the developing nervous system differentiate into distinct identities and is heavily controlled by several extracellular factors and signaling gradients across the center axis of the nervous system (dorso-ventral, antero-posterior, left-right). Thus, cell polarity is essential for proper asymmetric divisions, leading to neurogenesis, neuronal positioning, and cellular differentiation [93,94]. Many of the driving pathways of neurogenesis, such as Notch, Hh, and Wnt, have been directly connected to primary cilia [89,95,96,97,98,99]. While the phenotypes in ciliopathies are often heterogeneous, there are common pleiotropic features that include brain malformations and neurological impairment; although specifics vary in frequency and phenotype depending on the ciliopathy in question [100]. Cilia are essential in regulating progenitor differentiations, neural stem cell migration, and cerebrospinal fluid (CSF) movement [101]. Motile cilia function as locomotion for the movement of fluid in tissues, and while an in-depth dive into motile cilia is beyond the scope of this review, note that they play a fundamental role in the nervous system. Multiciliated cells are only present in a small portion of the population of cells in the brain and are dominantly responsible for the flow of CSF; a current that is important for the movement of nutrients and waste as well as the migration of neural progenitors [102]. The left-right asymmetry established early in vertebrate development is driven by monocilia known as nodal cilia, a special type of motile cilia that has the structure of primary cilia but rotates in a circular motion. This style of ciliary beating produces a left-ward current of fluid [102,103]. Research has shown to support two hypotheses for how this directional fluid movement impacts development: (1) The morphogen hypothesis; extracellular signaling molecules aggregate on the left side of the embryo in a sufficient concentration to trigger a signaling cascade, and (2) The two-cilia model; where mechanosensitive immotile primary cilia detect the current generated by nodal cilia, leading to a rise in intracellular Ca^2+^ and activating “left-sided” genes [104]. Interestingly, the common ground between these hypotheses is that there is an asymmetric Ca^2+^ gradient, but only the two-cilia hypothesis provides an explanation for how this signal is generated and transduced [105]. The two-cilia model came about after the discovery of mechanosensitive polycystins in PKD, when studies on development reported that PC-2 was found on both the nodal and primary cilia. Furthermore, mutations and knockout studies of PC-2 lead to a uniform Ca^2+^ distribution in the embryo, as well as a disruption in left-right patterning. This suggests that asymmetric Ca^2+^ is important for proper patterning and is mediated by PC-2 [68,69].

In addition to the flow of CSF fluid generated by motile cilia, spontaneous Ca^2+^ fluctuations have been shown to be important for neural migration along with other regulated developmental events, such as axonal outgrowth and maturation of signaling properties [106,107,108,109,110,111]. Additionally, embryonic stem cell-derived neural progenitors were found to form networks with concurrent oscillating Ca^2+^ activity that stimulated proliferation [112]. While sources of these Ca^2+^ signals may be due, in part, to standard cytoskeletal or cell-cell based communication, with the evidence presented above, as well as the mounting experimental and literary data not addressed here, primary cilia could play a significant part in mediating these essential Ca^2+^ fluctuations. While there is little investigation into the link between early neuronal Ca^2+^ signaling and primary cilia, there is also no evidence implicating any other structure or pathway in causing these oscillating Ca^2+^ levels. On studies performed on ventral spinal neurons during development in vitro, many of the proposed mechanisms for the oscillating Ca^2+^ influxes were ruled implausible [113]. The possibility of ionotropic AMPA (α-amino-3-hydroxy-5-methyl-4-isoxazolepropionic acid), NMDA (N-Methyl- d-aspartate), GABA (gamma aminobutyric acid), and glycine receptors being responsible for these fluctuations were ruled out due to the use of their respective receptor antagonists within the study. Any contribution by group I metabotropic glutamate receptor activity was shown to be minimal, and GABAergic and glycinergic Cl^−^-mediated transmission did not control these individual Ca^2+^ transients [113]. These spontaneous oscillations strongly depend on extracellular Ca^2+^ and require the ion’s entry through Ca^2+^ channels in the plasma membrane, a function that primary cilia are proposed to moderate in other cell types. L- and T-type channels were also not singularly responsible for these oscillations either, as their pharmacological block with Ni^2+^ or nifedipine only slowed down these events [113]. A mystery pathway dependent on the entry of Ca^2+^ to re-supply drained intracellular stores was proposed, and then was quickly proved unlikely when the use of thapsigargin, a potent blocker of this transport system, had no effect on the Ca^2+^ oscillations [114,115]. The use of apamin, a toxin which selectively blocks Ca^2+^ activated SK (small conductance Ca^2+^-activated potassium) channels, also had no apparent effect on these oscillations [116]. Through process of elimination, and homology with other primary cilia that do play Ca^2+^ signaling roles in their respective cell types, these neuronal primary cilia could play a significant role in mediating these Ca^2+^ oscillations.

### 3.4. Glucose-Mediated Insulin Secretion

Pancreatic islets regulate glucose homeostasis through the secretion of various peptide hormones and are comprised of β-cells, which interact with each other through both paracrine and autocrine mechanisms in order to serve this function [117]. β-cells control insulin secretion through a wide variety of interacting factors including the aforementioned autocrine and paracrine signaling, as well as cell–cell communication and neuronal, cellular, and vascular regulation [117]. β-cells contain primary cilia which regulate these processes though several different pathways; Ca^2+^ signaling mainly occurs in the autocrine insulin secretion pathway and will therefore be the focus of this section [117,118]. Because primary cilia regulate insulin secretion, a high incidence of diabetes is observed in certain ciliopathies, such as Bardet–Biedl and Alström syndromes [118].

In normal functioning pancreatic islets, in response to a glucose-mediated cytosolic Ca^2+^ increase, insulin receptors are recruited to the primary cilia [117]. The sequence of insulin secretion occurs as follows; an initial influx of Ca^2+^ triggers the first-phase insulin secretion, then a period of continuous Ca^2+^ oscillations triggers the second-phase insulin secretion [119,120]. Experiments done in mice with cilia-less β-cells (βCKO) show a complete failure of this sequence due to a loss of normal islet Ca^2+^ dynamics [118]. The cascading Ca^2+^ currents typically observed after glucose stimulation were abolished in the cilia-less βCKO line, leading to impaired insulin secretion, which established that glucose-mediated Ca^2+^ oscillations are cilia-dependent [118]. While the exact mechanism of Ca^2+^ regulation by β-cell primary cilium is unclear, the existence of Ca^2+^ channels on the ciliary membrane, as well as the Ca^2+^-fluxes serving as moderators of cytosolic Ca^2+^ levels are strong evidence of its vital role in glucose-mediated insulin secretion [11,42,121].

### 3.5. Biliary Regulation

When discussing primary cilia, often the sensory roles are looked at in isolation; in the kidneys they are mechanosensors, in the nose they are chemosensors. This, however, is not always an accurate depiction of just how vital it is for primary cilia to have its their unique multi-sensory functions. Much like most lumens, primary cilia can be found extending into the bile duct from cholangiocytes. Here, primary cilia simultaneously use all their sensory properties to tightly regulate luminal tonicity, biliary composition, and bile flow [67,74,122]. Fine-tuning the feedback loops necessary for proper bile secretion requires a number of ciliary-localized proteins. As bile modification by cholangiocytes is a highly coordinated process mediated by hormones, peptides, bile acids, and other molecules, the cholangiocyte cilia play a chemosensitive role in detecting the appropriate ligands in the extracellular environment and translating that signal into a response [123,124]. For example, the hormone secretin stimulates bile duct secretion, thereby increasing bile flow. The elevation in fluid flow would lead to ciliary bending and the mechanosensitive activation of the polycystin complex, PC-1/PC-2, generating a signal to stop bicarbonate secretion and halting fluid expansion. Simultaneously, TRPV4 is involved in the osmoregulation of bile as its activation and inhibition is dependent on the tonicity of bile. The activation of either or both the polycystin complex and TRPV4, as stated above, leads to an influx of Ca^2+^ which in general has varying effects on bile secretion [1,67,74].

### 3.6. Bone Formation

Mechanotransduction, in general, is critical for proper bone and cartilage homeostasis as its ability to adapt to its external environment is partly due to the fluid-saturated and porous nature of the tissue [125]. Reduced mechanical loading, or disuse of bone tissue, will lead to a decrease in bone density whereas regular mechanical loading will lead to an increase in bone density and strength [126]. Many mechanisms have been presented for how bone cells sense and translate physical signaling into a biochemical response, including fluid-shear stress through primary cilia. Osteocytes are considered the primary mechanosensors within bone and can be found in the fluid filled voids, dubbed lacunae, in the bone matrix [125]. The primary cilia extend from these cells and detect variation in fluid flow within the lacunar-canalicular networks caused by mechanical strain on the bones, often leading to bone remodeling [125,126]. While studies have demonstrated that the removal of osteocyte primary cilia disrupts fluid flow-induced osteogenic responses, a direct connection between ciliary fluid flow detection and bone matrix formation is still being established [75,127]. In vitro, evidence suggests that oscillatory fluid flow is required for the Ca^2+^ and mineral deposition associated with bone formation, a mechanism attributed to intact primary cilium with properly localized polycystin complexes and several TRP channel family proteins [73,75]. The opening of TRPV6 (Transient Receptor Potential Cation Channel Subfamily V Member 6) channels mediates a rise in intracellular Ca^2+^, which triggers cellular responses. When researchers pharmacologically inhibited Ca^2+^ channels, the osteocytes’ ability to respond to mechanical cues was impaired, and in vivo treatment with Ca^2+^ channel inhibitors was found to reduce skeletal responses to mechanical forces. ATP also increased upon mechanical stimulation, and several in vitro studies demonstrated that intracellular Ca^2+^ is required for this ATP response. However, the exact mechanism by which Ca^2+^ controls ATP release is still not completely understood [128]. Kif3a (Kinesin Family Member 3A), a motor protein important to cilia formation and function that has been shown to interact with PC-2 as well as to mediate proper ciliary polycystin localization, has been shown to have a role in osteoblast mechano-functions [70]. Osteoblasts derived from Kif3a knockout mice displayed lower basal cytosolic Ca^2+^ levels and had impaired intracellular Ca^2+^ responses to fluid-shear stress, as well as ciliary structural aberrations like a reduction in cilia length and number. In vivo, Kif3a null mice display lower bone density, altered bone volume, reduced bone formation rates, and impaired mechanical properties; all of which supports the idea that osteoblast function may be mediated through ciliary polycystins [71]. Despite this, there is evidence to suggest that primary cilia and/or the polycystins in bone do not response to fluid flow like renal cilia, and are in fact not responsible for mediating an intracellular Ca^2+^ influx but instead translate the signal through another, as of yet unidentified pathway [127]. One proposed alternative pathway involves ciliary-localized integrins that are able to detect small tissue deformations independent of ciliary deflection. While these studies were done in chondrocytes, this pathway has been presented as an explanation for a Ca^2+^-independent pathway in osteocytes [126]. 

## 4. Conclusions

Calcium cations are highly diverse secondary messengers present in a vast number of signaling pathways within the body. Because of its versatility, Ca^2+^ is often involved in cilia-dependent biological processes. Primary cilia have become recognized as being signaling powerhouses of the cell, and their signaling is often dependent on the presence of Ca^2+^. Depending on the organ system, primary cilia act as mechanosensors, chemosensors, electrosensors, or any combination of these. Working together, Ca^2+^ and cilia drive a vast array of pathways and processes within the body. The cell cycle, cell polarity and migration, neuronal patterning, glucose-mediated insulin secretion, biliary regulation, and bone formation are just some of the known and proposed ways that Ca^2+^ and cilia work together to maintain overall homeostasis. While the dysfunction of cilia and wayward Ca^2+^ signaling causes an ever-growing list of diseases, known as ciliopathies, the true extent of Ca^2+^ and cilia interactions is still poorly understood, yet likely more intertwined and prevalent than previously thought.

## Figures and Tables

**Table 1:** Tabular summary of ciliary Ca^2+^ channels and GPCRs and their functional role in various organ systems.

Ion Channel, GPCR, or Protein	Property	Functional Response	Citation
PC-1	Mechanosensative Membrane-Bound Protein, Possible Atypical GPCR	Vascular endothelial cells: activation of PC-1/PC-2 complex triggers CaM, PKC, and AKT/PKB, which in turn trigger eNOS, leading to NO production and subsequent vasodilationCell cycle: initially activates PC-2, which activates Ca^2+^/CaM-dependent pathways, then tail is cleaved and translocated to the nucleus to regulate DNA transcriptionCholangiocytes: involved in biliary regulation with PC-2, generates signals that modulate bile secretion based on external stimuli	[30,65,66,67]
PC-2	Ca^2+^-permeable Non-selective TRP Cation Channel	Vascular endothelial cells: activation of PC-1/PC-2 complex triggers CaM, PKC, and AKT/PKB, which in turn trigger eNOS, leading to NO production and subsequent vasodilationCell cycle: allows Ca^2+^ influx, which activates Ca^2+^/CaM-dependent pathwaysNeuronal patterning: allows for the asymmetrical Ca^2+^ distribution needed for left-right patterning Cholangiocytes: involved in biliary regulation with PC-2, generates signals that modulate bile secretion based on external stimuliOsteocytes: involved in osteoblast mechano-functions, possibly along with Kif3a	[30,65,67,68,69,70,71]
CaM	Ca^2+^-binding Messenger Protein	Vascular endothelial cells: activation of PC-1/PC-2 complex triggers CaM, which triggers eNOS, leading to NO production and subsequent vasodilationCell cycle: modulates Ca^2+^/CaM-dependent kinases I, II, and IV	[30,60]
TRPV2	Ca^2+^-permeable Non-selective TRP Cation Channel	Cell cycle: allows for an isolated rise in intraciliary Ca^2+^	[72]
TRPC1	Ca^2+^-permeable Non-selective TRP Cation Channel	Cell cycle: allows for an isolated rise in intraciliary Ca^2+^	[72]
TRPV4	Ca^2+^-permeable Non-selective TRP Cation Channel	Osteocytes: modulates Ca^2+^ levels, possibly in response to mechanical forcesCholangiocytes: osmoregulation of bile	[67,73,74]
TRPV6	Ca^2+^-permeable Non-selective TRP Cation Channel	Osteocytes: modulates Ca^2+^ levels, possibly in response to mechanical forces	[73,75]
Kif3a	Kinesin-like Protein	Osteocytes: involved in bone formation and osteoblast mechano-functions, possibly along with PC-2	[70,71]

## Abbreviations

Ca^2+^	Calcium cation
PC-2	Polycystin 2
TRPV4	Transient receptor potential cation channel subfamily V member 4
Wnt	Wingless-related integration site
PDGF	Platelet-derived growth factor
Hh	Hedgehog
PKD	Polycystic kidney disease
PC-1	Polycystin 1
NO	Nitric oxide
Ca^2+^/CaM	Calcium/calmodulin complex
eNOS	Endothelial nitric oxide synthase
CaM	Calmodulin
PKC	Protein kinase C
AKT/PKB	Protein kinase B
OSN	Olfactory sensory neurons
GPCR	G-protein-coupled receptor
CNG	Cyclic nucleotide-gated channel
CaMKII	Calmodulin-dependent protein kinase II
CP110	Centriolar coiled-coil protein of 110 kDa
TRPV2	Transient receptor potential cation channel subfamily V member 2
TRPP2	Transient receptor potential polycystin 2
TRPC1	Transient receptor potential cation channel subfamily C member 1
TGFβ	Transforming growth factor beta
CSF	Cerebrospinal fluid
AMPA	α-amino-3-hydroxy-5-methyl-4-isoxazolepropionic acid
NMDA	N-Methyl-d-aspartate
GABA	Gamma aminobutyric acid
Cl^−^	Chlorine ion
Ni^2+^	Nickel (II) ion
SK	Small conductance calcium-activated potassium channel
βCKO	Cilia-less β-cell line
TRPV6	Transient receptor potential cation channel subfamily V member 6
Kif3a	Kinesin family member 3A
